# Considerations for assessing the impact of the COVID-19 pandemic on mental health in Australia

**DOI:** 10.1177/0004867420947815

**Published:** 2020-08-03

**Authors:** Eric J Tan, Denny Meyer, Erica Neill, Andrea Phillipou, Wei Lin Toh, Tamsyn E Van Rheenen, Susan L Rossell

**Affiliations:** 1 Centre for Mental Health, Faculty of Health, Arts and Design, Swinburne University of Technology, Melbourne, VIC, Australia; 2 Department of Mental Health, St Vincent’s Hospital − Melbourne, Melbourne, VIC, Australia; 3 Department of Psychiatry, University of Melbourne, Melbourne, VIC, Australia; 4 Department of Mental Health, Austin Hospital, Melbourne, VIC, Australia; 5 Melbourne Neuropsychiatry Centre, Department of Psychiatry, University of Melbourne and Melbourne Health, Melbourne, VIC, Australia

**Keywords:** Coronavirus, COVID-19, mental health

## Abstract

During this unprecedented novel coronavirus (COVID-19) pandemic, there is an urgent need for empirical data to characterise its impact on the mental health and well-being of Australians. In this viewpoint, we outline a number of considerations for research on this topic, highlighting areas necessitating special attention, consideration of particular vulnerable groups and the need for longitudinal studies to track mental health fluctuations in the general population. We conclude by introducing the COLLATE (COvid-19 and you: mentaL heaLth in AusTralia now survEy) project, outlining its aims, addressing some considerations raised herein and detailing avenues for future research. Since the World Health Organization (WHO) declared the novel coronavirus (COVID-19) outbreak a Public Health Emergency of International Concern (PHEIC) on 30 January 2020 (WHO, 2020), the COVID-19 pandemic has caused major upheaval both in Australia and globally. While the search for a vaccine continues, current efforts towards tackling the virus and limiting contagion in several nations have focused on social distancing and the shutdown of non-essential services. In Australia, the first case was reported on 13 January 2020 (COVID-19 National Incident Room Surveillance Team, 2020), the first death occurred on 24 February and a spate of progressive restrictions were enforced throughout the 2 weeks leading up to 31 March 2020 (COVID-19 National Incident Room Surveillance Team, 2020a).

The rapid response to the COVID-19 pandemic that was demanded to minimise the spread of the virus led to some discrepant public messaging between the Australian Federal and State governments, with variations on the breadth of these restrictions between individual states (e.g. 16 legitimate reasons for leaving home in New South Wales vs only 4 in Victoria). Overall, the national message has been one of social distancing and minimal physical contact among members of different households. This was aimed at facilitating the now ubiquitous ‘flattening of the curve’ scenario, as a means of reducing the likelihood of healthcare system saturation and thereby unnecessary loss of life.

The swift government-enforced lifestyle changes will undoubtedly have had significant effects on Australians, with consequences for mental health and well-being at the forefront. For example, unemployment and being temporarily ‘stood down’ could contribute to a reduction in financial resources and subsequent pressures in the home situation. The era of 24-hour news cycles and ease of social media access confronts Australians with large amounts of often-negative and at times conflicting information. Given the expected extension of many of these changes and restrictions (e.g. social distancing, business closures, working from home), it is important to understand how the mental health and well-being of Australians are being affected, and how this may change over the course of the pandemic. In particular, there is an urgent and critical need to identify if there are certain groups of individuals that may require immediate, particular and sustained support.

Recently published data from COVID-19 studies in China have revealed significantly higher levels of anxiety (Wang et al., 2020), along with increased negative emotions (e.g. depression) and decreased positive emotions (e.g. happiness) in the general population (Li et al., 2020). Medical health workers also demonstrated increased insomnia, anxiety, depression, somatisation and obsessive-compulsive symptoms when compared to non-medical (e.g. administration) health workers (Zhang et al., 2020b). In terms of risk factors for negative emotions, the following were identified: being female (Wang et al., 2020; Zhang et al., 2020a), living in rural areas, being in contact with COVID-19-positive patients (Zhang et al., 2020a), student status, specific physical symptoms and poor self-rated health (Wang et al., 2020). Some of these were corroborated and expanded on in a Spanish study, identifying risk factors associated with higher levels of distress and loneliness: being female, being younger, having negative self-perceptions about ageing, fewer positive emotions, lower quality of sleep and higher expressed emotion (Losada-Baltar et al., 2020).

In the Australian context, there is a critical need for population-level data on the psychological impact of the pandemic. This will aid the development of government policies and initiatives in relation to mental health that can best support the Australian population. Such information is also critical for future pandemic and crisis planning (Holmes et al., 2020). Digital research methods, such as online surveys, would be an ideal rapid data collection exercise in cyber-connected nations, such as Australia. Below, we detail some key research priorities that we believe are of immediate and general need.

## Key areas of focus for COVID-19 mental health research

The first consideration is the urgent need to characterise current levels of mental health and well-being in the general population. For example, levels of depression, anxiety, stress, loneliness, positive and negative affect, feelings of loss and life satisfaction in society need to be determined, as well as understanding how these have changed as a result of the pandemic. In addition, the assessment of risk factors for adverse mental health outcomes at the population level will help us understand where support should be targeted. To do this, it would be beneficial to capture a range of factors. At a socio-demographic level, variables such age, gender, employment type and status, family size, social network size, frequency of non-physical interactions, adaptability to new situations (e.g. working from home, online communication), rural/urban residence, any current or previous mental health diagnoses, and alcohol and substance use information would be valuable to collect. In terms of physical health, factors such as sleep duration and quality would be important, as would eating and exercise behaviours, and information relating to current physical health and medical conditions. Psychological constructs that could prove informative include personality traits, aberrant thinking, coping styles, resilience, self-efficacy and life satisfaction.

A second consideration is a specific focus on the mental health and well-being of particular groups with pre-existing conditions or situations that could render them more vulnerable to the impacts of the COVID-19 pandemic, for example, individuals with existing diagnoses of serious mental illness (e.g. schizophrenia, bipolar disorder, eating disorders) (Druss, 2020). There is evidence that current high-level stresses and traumatic experiences can result in symptom exacerbation and relapse in psychosis and mood disorders (Docherty et al., 2009; Kessler, 1997), while higher levels of anxiety are related to increased eating disorder symptoms (Costarelli and Patsai, 2012). Increased levels of loneliness and isolation brought about by social distancing restrictions, coupled with the existing stigma of living with a mental illness (Druss, 2020), will likely be detrimental to their mental health and will require more dedicated support.

Other vulnerable groups include older adults who live alone, carers of those with a mental illness or special needs, school-aged children (including adolescents) and their parents, frontline health workers, essential workers (e.g. police, supermarket workers, delivery drivers) and victims of domestic violence. It is important to uncover how these groups are being affected by the pandemic to inform approaches to assist them. In light of the impacts of the COVID-19 pandemic, the number of people seeking mental health support services is expected to increase and will thus require greater government investment. Related to this, evaluations of the efficacy of these increased investments will also be essential.

The final consideration is the need for longitudinal studies that track changes in mental health during and beyond the COVID-19 pandemic, as the impact of restrictions and social isolation will likely be evident for an extended period (Holmes et al., 2020). Indeed, in Taiwan, significant mental health consequences were observed during the severe acute respiratory syndrome (SARS) pandemic, with greater psychological distress in people over 60 and with higher levels of education, even after the crisis had resolved (Peng et al., 2010). Thus, primary concerns and related mental health impacts may evolve and should be captured as individuals adapt to a ‘new normal’. The term ‘new normal’ has been used when referencing the current situation we find ourselves in; it can also denote the period when Australia emerges from effective lockdown and ‘regular life’ begins to resume. Further tracking of the population time-stamped to announcements for significant nationwide government initiatives, such as additional funding for mental health services or the employment support payments, will be beneficial in assessing the potential impact and efficacy of these initiatives on Australians. It is therefore imperative that alongside cross-sectional studies, there are long-term studies to track changes in mental health and well-being, as the COVID-19 pandemic evolves.

## Other relevant considerations

While the national focus remains on the negative impacts of the pandemic, it is worthwhile considering and assessing potential unanticipated positives that may have emerged. Indeed, a review conducted after the 2009 influenza A (H1N1) epidemic in Mexico revealed an increased awareness and uptake of personal hygiene practices nationwide in the wake of the virus (Córdova-Villalobos et al., 2009). Furthermore, after the SARS outbreak in Hong Kong, many residents reported feeling closer to their family and friends, and more in touch with their mental health, which led to them increasing time devoted to relaxation and exercising (Lau et al., 2006). In Australia and abroad, anecdotal evidence from news and social media has already highlighted potential silver linings associated with spending more time at home, including more time to do jobs around the house, and time to do more reading, exercise and enjoy hobbies, such as cooking. The use of simple open-ended questions about the personal experience of unexpected positives from the current situation would be one way to examine this and ensure a holistically understanding the impact of the COVID-19 pandemic.

Another point relates to the language used in research projects, particularly during this sensitive time. While it is perhaps inevitable that the bulk of study content may be negative (whether in questions or elicited responses), it is important that the questions asked are staggered by valence and worded neutrally where possible. This would help to prevent the unnecessary development of an overtly negative feel to the research, which may influence responses. It may also be beneficial to end on a positive note, for example, with a more encouraging questionnaire (e.g. a resilience measure). Language and phrasing can be a powerful and stigmatising tool (Tan, 2020), and in these uncertain times, it is contingent on researchers to be more sensitive to the prevailing circumstances in the interests of both respondent welfare and data quality.

It is conceivable that the impacts of the COVID-19 pandemic will be felt in existing mental health research and may affect a priori hypotheses and observed results. Consequently, it would be useful to incorporate questions relating to changes an individual may be experiencing or may have experienced because of the pandemic where possible. Potential free-response questions could include ‘Have there been significant changes in your life situation related to the COVID-19 pandemic?’, ‘How has your mental health and well-being been affected by the COVID-19 pandemic?’ and ‘Have you been affected by any changes in access to healthcare related to the COVID-19 pandemic?’ It might also be beneficial to include Likert-type rating options (e.g. not at all, moderately, significantly) to better quantify responses and permit such influences to be accounted for in later data analyses.

## The COLLATE project

To address a large number of these considerations, and with an urgent need to gather empirical data on the mental health impacts of the COVID-19 pandemic, we launched the COLLATE (COvid-19 and you: mentaL heaLth in AusTralia now survEy) project on 1 April 2020. Ethical approval was obtained from the Swinburne University Human Research Ethics Committee (approval number: 20202917-4107). The project includes a series of online surveys commencing at the start of each month and open for 72 hours. The surveys will run for 12 months (until March 2021), with four annual surveys thereafter, concluding in April 2024. Participants have and will continue to be recruited nationwide. They will be invited to complete as many or as few of the surveys as they wish, with the overall project seeking to gather snapshots of Australian mental health and well-being longitudinally. The data collected will be an invaluable resource towards not only an understanding of the current state of mental health and well-being in Australia, but also long-term trajectories. A number of measures form the core data collected, including socio-demographics such as age, gender, employment type and status, postcode (for identification of region and state), history of physical and mental health conditions, as well as measures of mood (depression, anxiety, stress, current positive and negative affect) (Watson et al., 1988), self-reported cognitive function and life satisfaction.

The COLLATE project team is committed to maximising the generalisability of the findings to the Australian population. We envision that the composition of respondents and response rates for each survey will differ over the life of the project. Consequently, when analysing survey results, we will adopt weighting of responses for variables such as age, gender and state/territory against published results from the most recent Australian Bureau of Statistics census (ABS, 2016: at present), where appropriate, to maximise the representativeness of results and comparability of data across different time points. When matched to prevailing and significant pandemic-related global and local events, the COLLATE surveys will provide potential markers of national mental health, as Australia navigates these uncharted times.

As Australia adapts to the ‘new normal’, key issues facing the population and their immediate concerns may quantitatively and qualitatively differ across time, and it is important to capture this information as it emerges. The flexibility of our ‘snapshot’ study design will allow the COLLATE project to metamorphose between survey iterations to better capture the effects of evolving situations and events as they happen. This could occur, for example, through the introduction of relevant new questions at later stages of the project (e.g. when restrictions are lifted) to gauge those specific impacts. We consider the adaptability of individual survey iterations within the COLLATE project to be a strength of the design. Coupled with the 4-year duration of assessment, the COLLATE project will be well-positioned to empirically assess the breadth of the COVID-19 pandemic and beyond. The project can be found on Facebook at ‘The Collate Project’ and via our Twitter handle: @collateproject.

Presently, the COLLATE project is gathering broad information from adults aged 18 years and above in the general population. There is clear scope for targeted adult populations, involving healthcare workers, the police, educators, specific mental health populations, carers and older adults. It would be beneficial to conduct international comparisons (Holmes et al., 2020) to explore the influence of cultural perspectives, as well as different virus mitigation strategies (e.g. widespread restrictions vs limited to no restrictions) on mental health. The COLLATE project team are open to collaborators who can aid in expanding the reach of future survey iterations during the life of the project and invite potential new stakeholders to contact the corresponding author. The COLLATE project presents a time-sensitive opportunity to provide critical, urgent and definitive information for community, policy and national benefit. We are optimistic that it will contribute significantly to the collegiate repository of COVID-19 and related mental health findings in Australia and around the world.

## References

[bibr1-0004867420947815] COVID-19 National Incident Room Surveillance Team (2020) 2019-nCoV acute respiratory disease, Australia Epidemiology Report 1: Reporting week 26 1 – 1 2 2020 Communicable Diseases Intelligence 44 DOI: 10.33321/cdi.2020.44.13. 32027812

[bibr2-0004867420947815] Australian Bureau of Statistics (ABS) (2016) ABS 2016 Census Table Builder. Available at: www.abs.gov.au/websitedbs/censushome.nsf/home/tablebuilder (accessed 13 July 2020).

[bibr3-0004867420947815] Córdova-Villalobos JA Sarti E Arzoz-Padrés J, et al (2009) The influenza A(H1N1) epidemic in Mexico. Lessons learned. Health Research Policy and Systems 7: 21. 19785747 10.1186/1478-4505-7-21 PMC2765941

[bibr4-0004867420947815] Costarelli V Patsai A (2012) Academic examination stress increases disordered eating symptomatology in female university students. Eating and Weight Disorders – Studies on Anorexia, Bulimia and Obesity 17: e164–e169. 10.1007/BF03325343 23086251

[bibr5-0004867420947815] COVID-19 National Incident Room Surveillance Team (2020a) COVID-19, Australia: Epidemiology report 10: Reporting week ending 23:59 AEST 5 4 2020 Communicable Diseases Intelligence 44 DOI: 10.33321/cdi.2020.44.30. 32268855

[bibr6-0004867420947815] Docherty NM St-Hilaire A Aakre JM, et al (2009) Life events and high-trait reactivity together predict psychotic symptom increases in schizophrenia. Schizophrenia Bulletin 35: 638–645. 18245057 10.1093/schbul/sbn002 PMC2669571

[bibr7-0004867420947815] Druss BG (2020) Addressing the COVID-19 pandemic in populations with serious mental illness. JAMA Psychiatry. Epub ahead of print 3 4 DOI: 10.1001/jamapsychiatry.2020.0894. 32242888

[bibr8-0004867420947815] Holmes EA O’Connor RC Perry VH, et al (2020) Multidisciplinary research priorities for the COVID-19 pandemic: A call for action for mental health science. The Lancet Psychiatry 7: 547–560. 32304649 10.1016/S2215-0366(20)30168-1 PMC7159850

[bibr9-0004867420947815] Kessler RC (1997) The effects of stressful life events on depression. Annual Review of Psychology 48: 191–214. 10.1146/annurev.psych.48.1.191 9046559

[bibr10-0004867420947815] Lau JT Yang X Tsui H, et al (2006) Positive mental health-related impacts of the SARS epidemic on the general public in Hong Kong and their associations with other negative impacts. Journal of Infection 53: 114–124. 16343636 10.1016/j.jinf.2005.10.019 PMC7132442

[bibr11-0004867420947815] Li S Wang Y Xue J, et al (2020) The impact of COVID-19 epidemic declaration on psychological consequences: A study on active Weibo users. International Journal of Environmental Research and Public Health 17: 2032. 10.3390/ijerph17062032 PMC7143846 32204411

[bibr12-0004867420947815] Losada-Baltar A Jiménez-Gonzalo L Gallego-Alberto L, et al (2020) ‘We’re staying at home’. Association of self-perceptions of aging, personal and family resources and loneliness with psychological distress during the lock-down period of COVID-19. Journals of Gerontology. Series B, Psychological Sciences and Social Sciences. Epub ahead of print 13 4 DOI: 10.1093/geronb/gbaa048. PMC7184373 32282920

[bibr13-0004867420947815] Peng EY Lee MB Tsai ST, et al (2010) Population-based post-crisis psychological distress: An example from the SARS outbreak in Taiwan. Journal of the Formosan Medical Association 109: 524–532. 20654792 10.1016/S0929-6646(10)60087-3 PMC3106105

[bibr14-0004867420947815] Tan EJ (2020) Having schizophrenia is not being ‘schizophrenic’: Your words matter. Australian and New Zealand Journal of Psychiatry 54: 210. 10.1177/0004867419877958 31552749

[bibr15-0004867420947815] Wang C Pan R Wan X, et al (2020) Immediate psychological responses and associated factors during the initial stage of the 2019 coronavirus disease (COVID-19) epidemic among the general population in China. International Journal of Environmental Research and Public Health 17: 1729. 10.3390/ijerph17051729 PMC7084952 32155789

[bibr16-0004867420947815] Watson D Clark LA Tellegen A (1988) Development and validation of brief measures of positive and negative affect: The PANAS scales. Journal of Personality and Social Psychology 54: 1063–1070. 3397865 10.1037//0022-3514.54.6.1063

[bibr17-0004867420947815] World Health Organization (WHO) (2020) Statement on the Second Meeting of the International Health Regulations (2005) Emergency Committee Regarding the Outbreak of Novel Coronavirus (2019-nCoV). Available at: www.who.int/news-room/detail/30-01-2020-statement-on-the-second-meeting-of-the-international-healthregulations-(2005)-emergency-committeeregarding-the-outbreak-of-novel-coronavirus-(2019-ncov) (accessed 30 January 2020).

[bibr18-0004867420947815] Zhang K Zhou X Liu H, et al (2020a) Treatment concerns for psychiatric symptoms in COVID-19-infected patients with or without psychiatric disorders. The British Journal of Psychiatry 217: 351. 32270760 10.1192/bjp.2020.84 PMC7190394

[bibr19-0004867420947815] Zhang WR Wang K Yin L, et al (2020b) Mental health and psychosocial problems of medical health workers during the COVID-19 epidemic in China. Psychotherapy and Psychosomatics 89: 242–250. 32272480 10.1159/000507639 PMC7206349

